# The Lasting Impact of Childhood Food Insecurity on Depression Trajectories: The Role of Citizenship and Timing

**DOI:** 10.1093/geroni/igaf122.1447

**Published:** 2025-12-31

**Authors:** Wuyi Dong

**Affiliations:** University of Massachusetts Boston, Boston, Massachusetts, United States

## Abstract

Using 2011-2018 China Health and Retirement Longitudinal Study and the 2014 Life History survey, this study examines the association between childhood food insecurity (before age 17) and later-life depression trajectories among Chinese older adults. It further explores the role of the rural-urban hukou divide and the timing of food insecurity across three developmental stages—early childhood (ages 0–5), middle childhood (ages 6–12), and adolescence (ages 13–17) shaping this relationship. The final sample includes 27,235 observations from 6,818 respondents aged 50 and older with the experience. Growth curve models with age centered at 55 were used. 81.3% of respondents experienced childhood food insecurity, with higher prevalence among rural hukou holders (82.9%) than urban counterparts (75.1%). Regression analyses show that childhood food insecurity is significantly associated with higher depressive symptoms in later life (β = 2.14, p < .001) but not with changes in symptom growth over time among the overall sample. Stratified analyses reveal that only among rural hukou holders, food insecurity is significantly associated with higher depressive symptoms at age 55 (β = 3.06, p < .001), though symptom growth slowed with age (β = -0.21, p < .01). Further, among rural hukou holders, those exposed to food insecurity in adolescence (ages 13–17), a critical transition period marked by significant physical, emotional, and social development, exhibited higher depressive symptoms at age 55 (β = 2.95, p < .01) than those exposed in early childhood (0–5).

